# Association between abdominal adiposity and subclinical measures of left-ventricular remodeling in diabetics, prediabetics and normal controls without history of cardiovascular disease as measured by magnetic resonance imaging: results from the KORA-FF4 Study

**DOI:** 10.1186/s12933-018-0721-0

**Published:** 2018-06-12

**Authors:** Christopher L. Schlett, Roberto Lorbeer, Carolyn Arndt, Sigrid Auweter, Jürgen Machann, Holger Hetterich, Birgit Linkohr, Wolfgang Rathmann, Annette Peters, Fabian Bamberg

**Affiliations:** 1 Department of Diagnostic and Interventional Radiology, University Hospital Heidelberg, Heidelberg, Germany; 2 Institute of Clinical Radiology, Ludwig-Maximilian-University Hospital, Munich, Germany; 3 Department of Diagnostic and Interventional Radiology, University of Tuebingen, Hoppe-Seyler-Straße 3, 72076 Tuebingen, Germany; 4 Institute for Diabetes Research and Metabolic Diseases, Helmholtz Centre Tuebingen, Tuebingen, Germany; 5 German Centre for Diabetes Research, Tuebingen, Germany; 6 Department of Biometry and Epidemiology, German Diabetes Center, Duesseldorf, Germany; 7 Institute of Epidemiology, Helmholtz Zentrum München, German Research Center for Environmental Health, Neuherberg, Germany; 8 Institute for Cardiovascular Prevention, Ludwig-Maximilian-University Hospital, Munich, Germany; 9 German Center for Cardiovascular Disease Research, Munich, Germany

**Keywords:** Magnetic resonance imaging, Intra-abdominal fat, Fatty liver, Ventricular remodeling, Diabetes mellitus

## Abstract

**Objectives:**

Local, abdominal fat depots may be related to alterations in cardiac function and morphology due to a metabolic linkage. Thus, we aimed to determine their association with subtle cardiac changes and the potential interaction with hyperglycemic metabolic states.

**Methods:**

Subjects from the general population and without history of cardiovascular disease were drawn from the Cooperative Health Research in the Region of Augsburg FF4 cohort and underwent 3 T cardiac and body MRI. Measures of abdominal adiposity such as hepatic proton-density fat fraction [PDFF_hepatic_], subcutaneous (SAT) and visceral abdominal fat (VAT) as well as established cardiac left-ventricular (LV) measures including LV remodeling index (LVCI) were derived. Associations were determined using linear regression analysis based on standard deviation normalized predictors.

**Results:**

Among a total of 374 subjects (56.2 ± 9.1 years, 58% males), 49 subjects had diabetes, 99 subjects had prediabetes and 226 represented normal controls. Only subtle cardiac alterations were observed (e.g. LVCI: 1.13 ± 0.30). While SAT was not associated, increasing VAT and increasing PDFF_hepatic_ were independently associated with increasing LVCI (β = 0.11 and 0.06, respectively), decreasing LV end-diastolic volume (β = − 6.70 and 3.23, respectively), and decreasing LV stroke volume (β = − 3.91 and − 2.20, respectively). Hyperglycemic state did not modify the associations between VAT or PDFF and LV measures (interaction term: all p ≥ 0.29).

**Conclusion:**

In a healthy population, VAT but also PDFF_hepatic_ were associated with subclinical measures of LV remodeling without evidence for a modifying effect of hyperglycemic state.

## Introduction

Diabetes, particular type-2 diabetes, threatens the health of a large number of individuals and is associated with worse prognosis, mainly because of increased risk for adverse cardiovascular events [1, 2]. Beside patients with manifest diabetes, there is a relevant number of patients with impaired glucose metabolism who do not satisfy diabetes criteria, and who are considered as pre-diabetics since they often progress into type-2 diabetes and have a higher risk of cardiovascular events [3, 4]. Thus, risk assessment is a crucial objective in this cohort in order to identify patients who could benefit from prevention.

A potential risk marker as well pathophysiological interlink are local fat depots such as visceral abdominal fat adipose tissue (VAT) or hepatic steatosis given stepwise higher levels between normal, pre-diabetic and diabetic patients [5]. Beside storage of lipids, adipose tissue has pro-inflammatory characteristics by secreting cytokines [6, 7]. However, epidemiological evidence demonstrated that VAT and not subcutaneous adipose tissue (SAT) was specifically associated with cardiovascular risk factors and coronary heart disease [8]. Also evidence from previous studies indicated that VAT was associated with left-ventricular (LV) morphology and/or function [9–19], often superior and/or independently of SAT or body mass index (BMI) [17, 18]. This was shown in non-diabetic patients by Rider et al. [10] where an independent association of increased VAT with decreased LV function was observed. Neeland et al. [11] demonstrated a correlation of VAT and LV morphology in obese and non-obese patients independently of the diabetes status. A sub-study of the MESA cohort including 4364 subjects, both insulin resistance and waist-to-hip-ratio (WHR)—a rough surrogate for VAT, were associated with concentric LV remodeling, a precursor to heart failure, both independent of BMI [13]. In a smaller subgroup of the MESA study with available abdominal CT scan, direct measures of VAT by CT were also associated with concentric LV remodeling [14]. Park et al. [15] pointed out an independent and synergistic association of VAT and skeletal muscle mass on LV mass and function in a Korean cohort study and noted that study participants with insulin resistance had more VAT. Given the potential association of local fat depots, particular VAT, to LV remodeling but also to diabetes [20], an influence of the diabetic status on the association between VAT and LV remodeling is suggestive and needs to be further examined.

Thus, our primary aim was to study the association of VAT with measures of LV morphology and function in a population free of previously known cardiovascular disease, potentially independent of cardiovascular risk factors and other measurements of obesity. Our secondary aim was to determine whether these associations are affected by hyperglycemic metabolic state.

## Methods

### Study design and population

The study was designed as a case–control study nested in a prospective cohort from the “Cooperative Health Research in the Region of Augsburg” (KORA) in which subjects with diabetes, with prediabetes and controls recruited from the FF4 follow-up of the KORA S4 study underwent whole-body MR imaging. The study design, sampling method and data collection are described in detail elsewhere [5, 21]. Briefly, subjects were excluded if there was history of cardiovascular disease defined as validated/self-reported stroke, myocardial infarction or revascularization. In addition, subjects with non-MRI safe devices including e.g. cardiac pacemaker or implantable defibrillator, report of cerebral aneurysm clip or serum creatinine ≥ 1.3 mg/dL were excluded.

The study was approved by the institutional review board of the medical faculty of Ludwig-Maximilian University Munich and all participants provided written informed consent.

### Health assessment

Subjects of the KORA S4 cohort were re-examined between June 2013 and September 2014 at the KORA study center. An oral glucose tolerance test was administered to all participants who had not been diagnosed for type-2 diabetes. For the definition of pre-diabetes, the 1998 World Health Organization criteria were applied [22]. Subjects with prediabetes had an impaired glucose tolerance (IGT) as defined by a normal fasting glucose concentration and a 2-h serum glucose concentration measured by oral glucose tolerance test (OGTT) ranging between 140 and 200 mg/dL and/or impaired fasting glucose (IFG), as defined by a fasting glucose level between 110 and 125 mg/dL and a normal 2-h serum glucose concentration. Individuals with a 2-h serum glucose concentration measured by OGTT above 200 mg/dL and/or a fasting glucose level above 125 mg/dL were classified as newly diagnosed diabetics. Subjects with normal glucose metabolism with a 2-h serum glucose concentration measured by OGTT below 140 mg/dL and a fasting glucose level below 110 mg/dL were classified as normal controls.

Other established risk factors were collected in standardized fashion as part of the KORA study design and described elsewhere [5]. Briefly, hypertension was defined as systolic blood pressure of at least 140 mmHg or diastolic blood pressure of at least 90 mmHg or current antihypertensive treatment. Subjects were classified as smokers if they had smoked at least one cigarette per day in the year prior to the study. BMI was defined as weight (kg) divided by the height squared (m^2^). Medications were assigned as ‘antihypertensive medication’ only if the compounds taken were classified as antihypertensively effective by the most recent guidelines. Antithrombotic medication comprised anticoagulants and antiplatelet drugs. Lipid lowering medication was defined as treatment with statins, fibrates or other lipid modifying agents.

### Magnetic resonance imaging

MR images were acquired using a 3 T Magnetom Skyra (Siemens AG, Healthcare Sector, Erlangen Germany) equipped with a whole-body coiling system. All subjects underwent the imaging protocol within 3 months after the visit at the study center. The whole-body protocol is described in detail elsewhere [5]. All image analyses were performed in blinded fashion by independent readers unaware of the diabetic status and clinical covariates on dedicated off-line workstations.

### Assessment of abdominal adipose tissue by magnetic resonance imaging

VAT and SAT were estimated at the umbilical level on a single axial slice since this approach is representative for the total amount of abdominal adipose tissue [23]. The amount of abdominal fat was measured on an axial reconstructed 3D VIBE-Dixon image (5 mm slice thickness) in cm^2^ and segmented by an automated procedure based on fuzzy-clustering [24].

### Assessment of hepatic lipids by magnetic resonance imaging

For determination of hepatic lipid content, a multi-echo Dixon-VIBE sequence was used with 6 T (1.23, 2.46, 3.69, 4.92, 6.15, 7.38 ms) accounting for T2* decay and the spectral complexity of fat (slice thickness 4 mm) [25, 26]. Using OsiriX (Version 4.1), a manual region of interest was drawn at the level of the portal vein excluding the hilus and large vessels for estimation of Pearson’s correlation coefficients [5].

### Assessment of cardiac function and morphology by magnetic resonance imaging

The cine-SSFP sequences were evaluated semi-automatically using commercially available software (cvi42, Circle Cardiovascular Imaging, Calgary, Canada). Following automatic contour detection of the LV endocardium, all borders were corrected manually, if necessary. LV myocardial mass (LVM), LV end-diastolic volume (LVEDV), LV end-systolic volume (LVESV) and ejection fraction (LVEF) were derived accordingly to current guidelines [27]. LV concentricity index (LVCI) was calculated as ‘LVM/LVEDV’, an abnormal increased LVCI was defined > 1.3 g/mL [28]. LV stroke volume (LVSV) was calculated as ‘LVEDV–LVESV’. The parameters LVM, LVEDV, LVESV, and LVSV are indexed based on body surface area (BSA) for all analyses. LV hypertrophy was defined increased LVM (≥ 96 g/m^2^ [women] and ≥ 116 g/m^2^ [men]) [29]; eccentric vs. concentric LV hypertrophy was based on an abnormal LVCI.

### Statistical analysis

Subject demographics, cardiovascular risk factors and MR outcomes are presented for the overall study sample and according to VAT tertiles as means and standard deviations for continuous variables and counts and percentages for categorical variables. Measurement differences among VAT tertiles were evaluated by one-way ANOVA and χ^2^ test, respectively. Correlations between abdominal fat and LV measures were displayed by scatter plots and Pearson’s correlation coefficients were provided.

Associations of abdominal fat with LVM, LVCI, LVEDV and LVSV were assessed by separate linear regression models with β-coefficients and 95% confidence intervals (CI). Abdominal fat parameters were modelled as standard deviation increments. Regression models were adjusted (a) for age and sex, (b) for age, sex and BMI and (c) fully. For the fully adjusted model, covariates beyond age, sex and BMI were selected based on univariate analysis (Appendix Table 4; all with p < 0.10); the fully model included hypertension, diabetes, triglycerides, HDL (for all LV parameters), additionally LDL (for LVCI, LVEDV, and LVSV) and lipid lowering medication (for LVM, and LVCI). As a sensitivity analysis, the fully adjusted models were repeated with a fixed set of typical cardiovascular risk factors (including age, sex, BMI, hypertension, diabetes, and smoking status) and with a fixed set of typical cardiovascular risk factors replacing the definition of hypertension by actual measures of systolic and diastolic blood pressure and the presence of antihypertensive medication (including age, sex, BMI, systolic blood pressure, diastolic blood pressure, antihypertensive medication, diabetes, smoking).

Furthermore the conjoint associations of abdominal fat parameters with LVCI, LVEDV and LVSV were estimated by age, sex and BMI adjusted linear regression model. Forrest plots were drawn and model-fit was expressed by R^2^.

A multiplicative interaction effect of diabetes status (normal controls, prediabetic and diabetic subjects) on the association between VAT and LV measures was tested. In addition, associations between VAT and LV measures were separately analysed for the diabetes groups by age and sex adjusted linear regression models and by boxplots of LVCI across tertiles of VAT including a trend test.

A p value of < 0.05 was considered to indicate statistical significance. All analyses were conducted with Stata 14.1 (Stata Corporation, College Station, TX, USA).

## Results

A total of 400 subjects without clinically known cardiovascular disease underwent MR imaging and complete VAT and LV measurements were available in 374 subjects. Excluded subjects did not differ from the included subjects with respect to age, gender or diabetic status (all p ≥ 0.36). Of the final cohort (age 56.2 ± 9.1 years, 58% males), 49 subjects had diabetes, 99 subjects had prediabetes and 226 represented normal controls. Based on MRI measurements, mean VAT was 147.31 ± 85.02 cm^2^ with lower tertile from 11.06 to < 98.94 cm^2^, mid tertile from 98.94 to < 175.79 cm^2^ and upper tertile from 175.79 to 456.36 cm^2^. Demographic and risk profiles—stratified by VAT tertiles—are provided in Table 1. VAT was highly interlinked with other measures of adiposity (Table 1).

**Table 1:** Characteristics of the study sample according to VAT tertiles

	All subjects	VAT—lower tertile	VAT—mid tertile	VAT—upper tertile	p value*
N	374	124	125	125	
Age (years)	56.2 ± 9.1	51.8 ± 7.8	57.0 ± 9.3	59.8 ± 8.3	< 0.001
Sex (men)	57.8% (216)	35.5% (44)	61.6% (77)	76.0% (95)	< 0.001
BMI (kg/m^2^)	27.9 ± 4.8	24.2 ± 3.1	28.7 ± 4.1	30.9 ± 4.3	< 0.001
Diabetes status					
Normal	60.4% (226)	91.9% (114)	63.2% (79)	26.4% (33)	< 0.001
Prediabetes	26.5% (99)	6.5% (8)	28.8% (36)	44.0% (55)	
Diabetes	13.1% (49)	1.6% (2)	8.0% (10)	29.6% (37)	
HbA1c	5.6 ± 0.7	5.3 ± 0.4	5.6 ± 0.9	5.8 ± 0.8	< 0.001
Hypertension	33.4% (125)	13.7% (17)	30.4% (38)	56.0% (70)	< 0.001
Systolic RR (mmHg)	121 ± 17	110 ± 12	123 ± 16	129 ± 16	< 0.001
Diastolic RR (mmHg)	75 ± 10	70 ± 8	77 ± 10	79 ± 10	< 0.001
Antihypertensive medication	24.6% (92)	12.1% (15)	22.4% (28)	39.2% (49)	< 0.001
Triglyceride levels (mg/dL)	130.5 ± 84.3	83.9 ± 38.9	132.2 ± 80.9	175.0 ± 95.6	< 0.001
Total cholesterol (mg/dL)	217.6 ± 36.4	207.8 ± 33.9	225.9 ± 36.4	219.1 ± 36.8	< 0.001
HDL (mg/dL)	62.0 ± 17.5	69.8 ± 19.3	61.6 ± 14.4	54.7 ± 15.1	< 0.001
LDL (mg/dL)	139.4 ± 33.0	129 ± 30.5	147.6 ± 31.4	141.4 ± 34.5	< 0.001
Lipid lowering medication	10.4% (39)	3.2% (4)	9.6% (12)	18.4% (23)	< 0.001
Smoking status					
Never-smoker	36.1% (135)	41.9% (52)	36.0% (45)	30.4% (38)	0.03
Ex-smoker	43.9% (164)	33.1% (41)	44.8% (56)	53.6% (67)	
Current-smoker	20.1% (75)	25.0% (31)	19.2% (24)	16.0% (20)	
MRI-based adiposity measures					
VAT (cm^2^)	147.31 ± 85.02	57.8 ± 23.23	139.77 ± 22.56	243.64 ± 57.22	N/A
SAT (cm^2^)	278.51 ± 117.44	210.00 ± 86.43	308.84 ± 119.13	316.73 ± 113.14	< 0.001
PDFF_hepatic_ (%)	8.4 ± 8.4	2.8 ± 2.3	7.0 ± 6.7	15.5 ± 8.9	< 0.001
MR-based LV measures					
LV mass, indexed (LVM; g/m^2^)	71.7 ± 13.9	67.0 ± 11.8	72.5 ± 15.2	75.6 ± 13.2	< 0.001
LV concentricity index (LVCI; g/mL)	1.13 ± 0.30	0.94 ± 0.19	1.11 ± 0.23	1.33 ± 0.33	< 0.001
LV end-diastolic volume, indexed (LVEDV; mL/m^2^)	66.20 ± 14.87	72.52 ± 13.41	66.82 ± 13.65	59.3 ± 14.56	< 0.001
LV ejection fraction (LVEF; %)	69.2 ± 8.2	68.5 ± 8.6	69.4 ± 7.3	69.6 ± 8.6	0.53
LV stroke volume, indexed (LVSV; mL/m^2^)	45.4 ± 9.7	49.4 ± 9.6	45.9 ± 8.5	40.8 ± 9.0	< 0.001

Data are means and standard deviations for continuous variables and counts and percentages for categorical variables

*RR* blood pressure, *VAT* visceral adipose tissue, *SAT* subcutaneous adipose tissue, *PDFF*_*hepatic*_ hepatic proton-density fat fraction, *LV* left-ventricular

* p values are from one-way ANOVA and χ^2^ test, respectively

### LV morphology and function and its correlation with adiposity

Measures of LV morphology and function are provided in Table 1. In this low-risk population and based on the MRI-based LVM measurements, LV hypertrophy was observed in four subjects—two with concentric and two with eccentric remodeling. In addition, three subjects had increased LVCI levels with normal LVM.

**Fig. 1:**
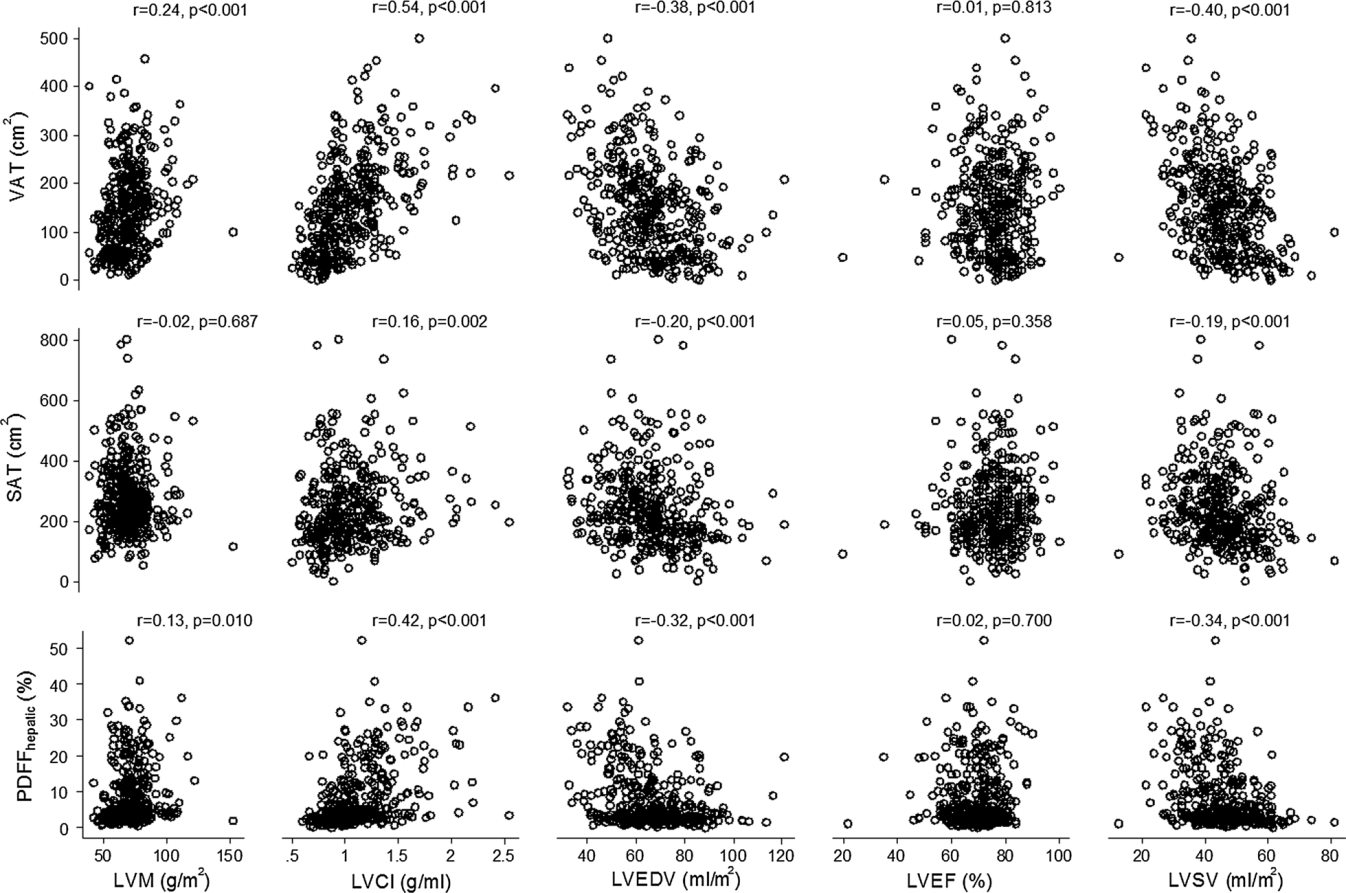
Correlation between abdominal fat depots and LV measures. Pearson’s correlation coefficients (r; together with p values) were provided for the correlation of the different fat depots including subcutaneous (SAT) and visceral (VAT) abdominal fat as well as PDFF_hepatic_

A stepwise increase was observed for LVM and LVCI across tertiles of VAT, while VAT decreased for LVEDV and LVSV (Table 1). LVEF was not different between VAT tertiles. Correlations between LV measures and MR-measures of adiposity are illustrated in Fig. 1; interestingly while for LVCI, LVEDV and LVSV all measures of adiposity were significantly correlated, only VAT was significantly correlated with LVM. Overall, strongest correlation was between VAT and LVCI (r = 0.54, p < 0.0001).

### Multivariable analysis of the association between abdominal adiposity and LV measures

The associations of VAT, SAT and PDFF_hepatic_ with LVM, which were observed in univariable analysis (Table 1), were attenuated after adjustment for age and gender (all p ≥ 0.21, Table 2). Further, none of the fat depots were associated with LVEF in any of the multivariate models (all p ≥ 0.14).

**Table 2:** Association of local abdominal fat depots with LV mass, volumes and function

	LVM		LVCI		LVEDV		LVSV	
	β (95% CI)	p	β (95% CI)	p	β (95% CI)	p	β (95% CI)	p
Separate models adjusted for age, sex								
VAT	0.78 (− 0.68; 2.23)	0.29	0.14 (0.11; 0.17)	< 0.001	− 6.79 (− 8.36; − 5.21)	< 0.001	− 4.26 (− 5.30; − 3.22)	< 0.001
SAT	0.83 (− 0.45; 2.10)	0.21	0.07 (0.04; 0.10)	< 0.001	− 2.98 (− 4.45; -1.50)	< 0.001	− 2.02 (− 2.99; − 1.05)	< 0.001
PDFF_hepatic_	0.37 (− 0.98; 1.71)	0.59	0.10 (0.07; 0.13)	< 0.001	− 4.75 (− 6.26; − 3.24)	< 0.001	− 3.15 (− 4.14; − 2.16)	< 0.001
Separate models adjusted for age, sex, BMI								
VAT	–		0.15 (0.11; 0.19)	< 0.001	− 7.92 (− 9.93; − 5.91)	< 0.001	− 4.8 (− 6.13; − 3.47)	< 0.001
SAT	–		0.01 (− 0.05; 0.06)	0.83	− 1.69 (− 4.72; 1.34)	0.28	− 1.42 (− 3.41; 0.58)	< 0.001
PDFF_hepatic_	–		0.09 (0.06; 0.12)	< 0.001	− 4.43 (− 6.10; − 2.76)	< 0.001	− 2.91 (− 4.00; − 1.81)	< 0.001
Separate, fully adjusted models^a^								
VAT	–		0.11 (0.07; 0.15)	< 0.001	− 6.70 (− 8.84; − 4.55)	< 0.001	− 3.91 (− 5.32; − 2.50)	< 0.001
SAT	–		–		–		− 1.75 (− 3.66; 0.16)	0.07
PDFF_hepatic_	–		0.06 (0.02; 0.09)	0.001	− 3.23 (− 5.03; − 1.44)	< 0.001	− 2.20 (− 3.37; − 1.04)	< 0.001

Separated models were fit for VAT, SAT and PDFF_hepatic_. β-coefficients represent change in LV parameters for standard deviation increment in abdominal fat measurements estimated by linear regression; ^a^ the fully adjusted model included age, sex, BMI, hypertension, diabetes, triglycerides, HDL (for all LV parameters), additionally LDL (for LVCI, LVEDV, and LVSV) and lipid lowering medication (for LVM, and LVCI). The selection of potential confounders for the fully adjusted model was done in univariate analyses for each of the different LV measurements (Appendix Table 4) to allow appropriate comparisons of the associations of the three fat depots to a particular LV measurement, but may limit the comparison between different LV measurements. To address this issue, sub-analyses were performed with a fixed set of common cardiovascular risk factors as potential confounders, no substantial differences were found (Appendix Table 5)

*VAT* visceral adipose tissue, *SAT* subcutaneous adipose tissue, *PDFF*_*hepatic*_ hepatic proton-density fat fraction, *LV* left-ventricular, *LVM* left-ventricular mass (in g/m^2^); *LVCI* left-ventricular concentricity index (in g/mL), *LVEDV* left-ventricular end-diastolic volume (in mL/m^2^), *LVSC* left ventricular stroke volume (in mL/m^2^)

In contrast, the association of VAT and PDFF_hepatic_ with LVCI, LVEDV and LVSV persisted in all models adjusting for all potential confounders (Table 2). While LVCI increased with increasing amount of fat, the association of LVEDV and LVSV were inversely associated with abdominal fat. Comparing VAT and PDFF_hepatic_ in the association to LV measures, the effect size per standard deviation of VAT was larger than for PDFF_hepatic_ throughout all LV measures and models.

Conversely, SAT was associated with LVCI and LVEDV in a basic model taking into account for age and gender, however attenuated by adjusting for BMI additionally. SAT and LVSV remained associated with LVDV also in a model including BMI and became borderline non-significant only in a fully adjusted model (p = 0.07). However, the effect size of SAT remained always below the effect size of PDFF_hepatic_ and even more below VAT (Table 2). These findings did not change substantially in sensitivity analysis for including different sets of potential confounders (Appendix Table 5).

**Fig. 2:**
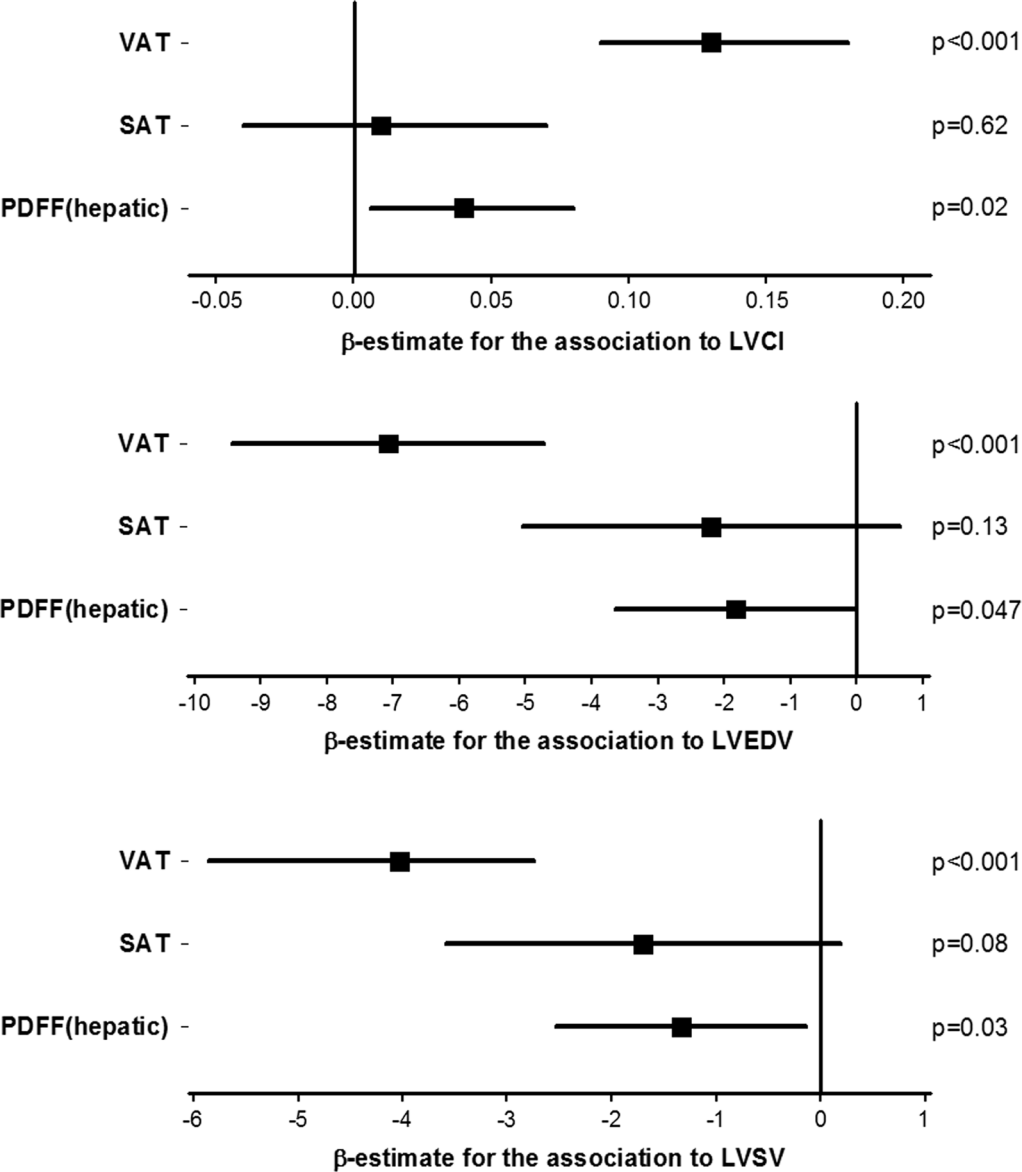
Forrest-plots of age-, gender- and BMI-adjusted model including VAT, SAT, and PDFF_hepatic_ for the Association to LVCI, LVEDV and LVSV. The model-fit of the different models expressed as r-square were 0.33, 0.23 and 0.21 for predicting LVCI, LVEDV and LVSV

In a model adjusting for age, gender, and BMI and all abdominal fat depots, both VAT and PDFF_hepatic_ provided independent and incremental value of predicting LVCI, LVEDV and LVSV (Fig. 2). While these six variables resulted in an r-square of 0.21 and 0.23 for LVSV and LVEDV respectively, the r-square was 0.33 for LVCI.

### Effect of the diabetic status on the observed associations

**Fig. 3:**
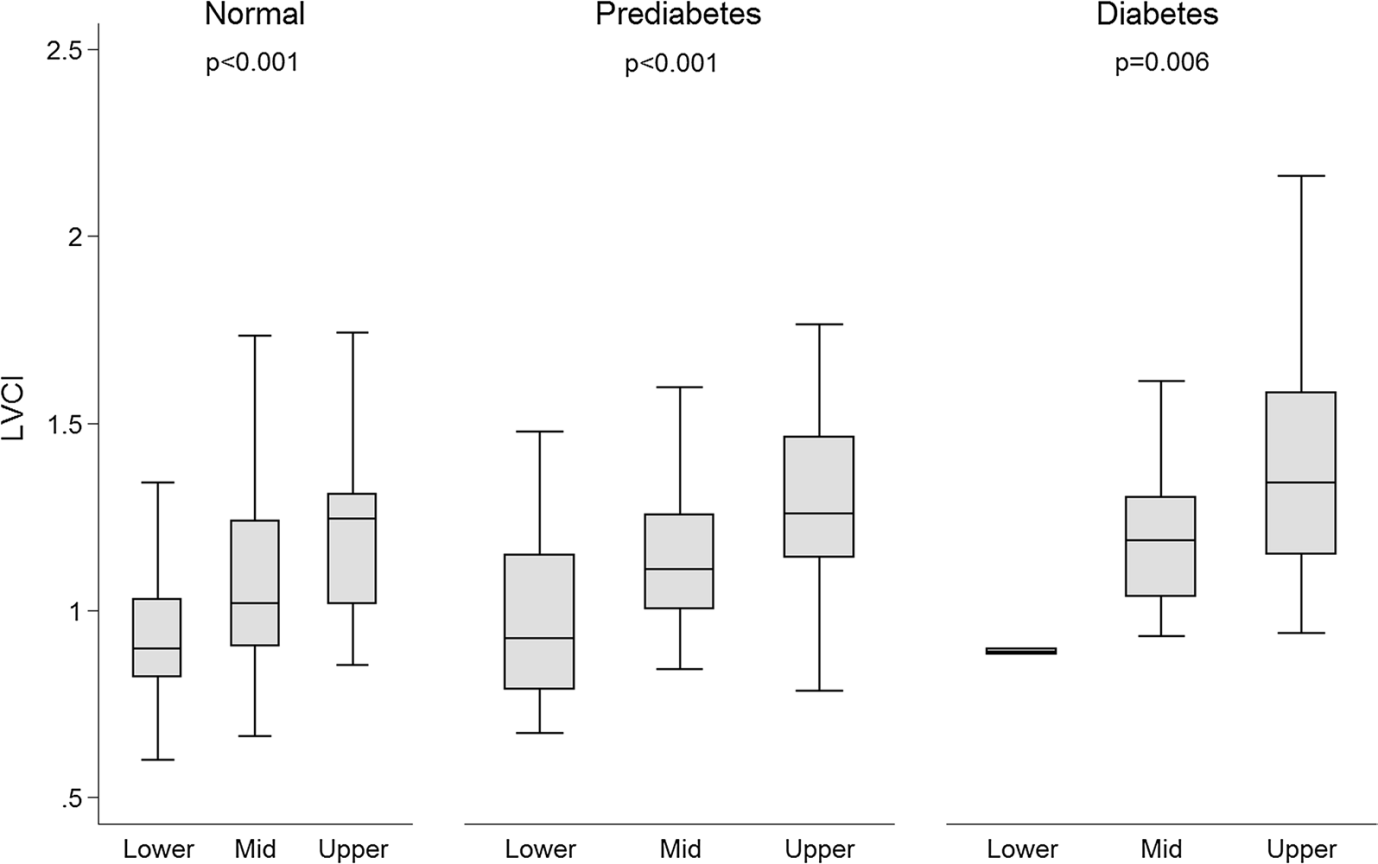
Subgroup-analysis stratified into subjects with diabetes, prediabetes and normal controls. Boxplots of left ventricular concentricity index (LVCI) across tertiles of abdominal visceral adipose tissue (VAT). p value represents a trend-test. (49 diabetes [lower: 2, mid: 10, upper: 37], 99 prediabetes [lower: 8, mid: 36, upper: 55] and 226 normal controls [lower: 114, mid: 79, upper: 33]

Assessing the association between VAT and LV measures in the subgroups of subjects with diabetes, prediabetes and controls, similar trends were observed as in the overall cohort, for example between VAT and LVCI (Fig. 3). The predefined interaction term to test whether the hyperglycemic metabolic status effects the association between VAT and LV measures was non-significant (all p ≥ 0.29; Table 3). Similarly, there was no effect modification between HbA1c-Levels and the association between VAT and LV measures (all p ≥ 0.12).

**Table 3:** Effect of diabetic status in the association of adiposity with LV parameters

	Controls	Pre-diabetics	Diabetics	p value inter-action
	β (95% CI)	β (95% CI)	β (95% CI)	
VAT—LVCI	0.11 (0.08; 0.15)	0.10 (0.03; 0.16)	0.16 (0.04; 0.28)	0.74
VAT—LVEDV	− 6.59 (− 8.81; − 4.36)	− 3.91 (− 7.05; − 0.77)	− 4.18 (− 9.7; 1.34)	0.77
VAT—LVSV	− 4.03 (− 5.62; − 2.44)	− 2.46 (− 4.4; − 0.53)	− 3.90 (− 6.86; − 0.94)	0.81

The β-coefficients represent change in LV parameters for one standard deviation increment in VAT estimated by linear regression (adjusted for sex, age); the models were not fit for the association between VAT and LVM since they were non-significant after adjustment for age and gender (Table 2)

*VAT* visceral adipose tissue, *LV* left-ventricular, *LVCI* left-ventricular concentricity index (mL/g), *LVEDV* left-ventricular end-diastolic volume (mL/m^2^), *LVSV* left ventricular stroke volume (mL/m^2^)

## Discussion

In this nested case–control MRI study from the prospective, population-based KORA cohort in subjects without history of cardiovascular disease, we observed an independent association of VAT and PDFF_hepatic_ to LVCI, a measure of early LV remodeling, but also to LVEDV and LVSV, a potential indicator for diastolic dysfunction. In contrast, no independent association was observed for abdominal adiposity to LVM nor for SAT to any of the LV measures. Furthermore, hyperglycemic metabolic state did not modify the association between abdominal adiposity and LV measures.

### Association of abdominal adiposity to LV remodeling

There is increasing evidence that abdominal adiposity is associated with worse metabolic state and cardiovascular complications [8]. Predominantly, this effect can be attributed to the amount of VAT as shown in a growing number of clinical and epidemiological studies, while SAT appears a more innocent bystander [11, 14, 15, 19, 29]. Our results add to the growing body of data by confirming an independent association for VAT to measures of LV remodeling, but not for SAT. Thus, our results support previous findings on the highly relevant role of VAT and extend these to a relatively large western European population.

LV remodeling index as the ratio between LVM and LVEDV is an important MR-based cardiac measure representing a precursor of heart failure and worse outcome [30]. Prior research by Abasi et al. [14], which was derived from the MESA cohort, similarly observed increased LV remodeling indices in subjects with higher VAT level. In line with the MESA cohort, our cohort also excluded history of cardiovascular disease. Their finding also demonstrates a subtle increase in LVCI, much smaller than observed in other studies, such as the Dallas heart study [11]. In a relatively small cohort of 75 nondiabetic men, Granér et al. [19] showed that hepatic triglyceride as measured by MR spectroscopy and VAT were associated with dedicated, echocardiography based measures of LV diastolic dysfunction. Diastolic dysfunction caused by hypertrophy in which filling is impaired due to low ventricular compliance may result in reduced LVEDV while both LVSV and LVEDV can be reduced without significant impact on LVEF. Accordingly, we found an association of abdominal adiposity with both, LVSV and LVEDV but not with LVEF, which may indicate that abdominal adiposity may affect more strongly the diastolic than systolic function in a first pathophysiologic step.

As described above, previous evidence on the association between hepatic steatosis and LV remodeling on a population-based cohort level is rare, given that most studies such as MESA or FHS employed CT to determine VAT and SAT and liver density in Hounsfield units by CT. However, CT represents only limited methods for assessment of adipose tissue content of the liver parenchyma [31] and it was only recently that rapid but robust and accurate multi-echo Dixon MR sequences became available [26]. As such, we demonstrate that MR-based PDFF_hepatic_ is independently and incrementally associated with subtle changes of LV remodeling, particular beyond VAT and independent of diabetes.

In a case–control study including 19 adults with type 2 diabetes, 19 adults with non-alcoholic fatty liver disease (NAFLD) and 19 healthy controls, Dr. Cassidy et al. [32] showed that changes in cardiac structure are related with both, diabetes and NAFLD, even without overt cardiac disease and without changes in cardiac energy metabolism. They postulated an interaction between NAFLD and diabetes with a two-hit hypothesis [32]. However, we couldn’t reveal any evidence for an interaction of diabetes with PDFF_hepatic_ regarding LV remodeling.

Beside overlapping risk factors for developing NAFLD and developing cardiovascular disease, there are several pathophysiological hypotheses of a more direct linkage [33]. NAFLD is associated with an atherogenic lipid profile with e.g. the increased production of triglyceride-rich very-low-density lipoprotein (VLDL) particles is increased [34]. Similar, the modification of cytokines including plasminogen activator inhibitor 1, adiponectin or interleukin 6, have been described in the association with NAFLD, and more strongly with non-alcoholic steatohepatitis [33]. Also, it has been shown that endothelial dysfunction occurs in experimental studies after a few days of high-fat feeding, when steatosis has developed but inflammation has not [35]. Nevertheless, indirect linkage of NAFLD to cardiovascular disease across NAFLD as a player in the development of diabetes and the metabolic syndrome must be recognized as well [33]. Thus, further study is hence needed to gain mechanistic insight into the pathophysiology of the hepatic steatosis and LV structural changes as well as cardiovascular disease.

### Metabolic connection between abdominal adiposity and LV remodeling

It is important to note that our cohort has a relatively limited size compared to MESA (n = 1151) or Dallas heart study (n = 2710). However, due to the nested design, our population had a higher percentage of patients with prediabetes (27%) and diabetes (13%) than the other two population-based cohorts (in MESA: 9 and 4% and Dallas heart study: N/A and 11%; respectively). Despite this increased power to detect difference, we did not reveal any interaction of the diabetic status on the correlation of VAT and LV-parameters even though Shah et al. and Canepa et al. [13, 18] suggested a metabolic connection between the interactions of VAT and LV parameters. Thus, our finding are in line with Rider et al. [10] who described insulin as an exemplary serum marker in diabetes for predicting LVM and Neeland et al. [11] who noted that the correlation between VAT and LV are independent of adipocytokines and insulin resistance. Also, our results add to the hypothesis by Shah et al. [13] that insulin resistance serves as a confounder in the interaction of obesity and LV remodeling as the correlation of BMI and LV parameters attenuated after multivariable adjustment (e.g. waist-to-hip-ratio). We confirm these observations by demonstrating that clearly VAT but not SAT is associated with subtle LV impairment. Similarly, a cardio-metabolic connection for NAFLD has been postulated by VanWagner et al. [36] As measured by CT, subclinical LV remodeling by echocardiography strain analysis from the multicenter, community-based coronary artery risk development in young adults (CARDIA) study was associated with LV parameters; however, in contrast, our data demonstrate that VAT is much more strongly associated with subclinical myocardial dysfunction as compared to PDFF_hepatic_. Clearly, further studies are needed to yield a better understanding of the metabolic connection of abdominal adiposity and cardiac parameters.

### Limitation

Our results do not imply causality because of the cross-sectional study design and need to be confirmed in longitudinal studies. Also, since the KORA cohort is single-centered, generalizability and external validity of our findings must be confirmed in the future. Because of the limited sample size and certain small subgroups (e.g. subjects with low VAT but presence of diabetes) we might lack statistical power to demonstrate significant interaction of diabetes in the association between VAT and LV remodeling.

## Conclusion

In conclusion, particularly VAT but also fatty liver parenchyma are independently and incrementally associated with early changes of LV remodeling in a general western population without history of cardiovascular disease. Although a metabolic connection is suggestive, no interaction with the diabetic status was revealed for these important associations.

## Appendix

See Tables 4 and 5.

**Table 4:** Selection of confounders based on univariate analysis

	LVM	LVCI	LVEDV	LVSV
Age (years)	*PreDef (0.33)*	*PreDef (< 0.001)*	*PreDef (< 0.001)*	*PreDef (0.001)*
Sex (men)	*PreDef (< 0.001)*	*PreDef (< 0.001)*	*PreDef (0.24)*	*PreDef (0.33)*
BMI (kg/m^2^)	*PreDef (0.007)*	*PreDef (< 0.001)*	*PreDef (< 0.001)*	*PreDef (< 0.001)*
Diabetes status	*0.03*	*< 0.001*	*< 0.001*	*< 0.001*
HbA1c	0.003*	< 0.001*	0.004*	< 0.001*
Hypertension	*< 0.001*	*< 0.001*	*0.02*	*0.08*
Systolic RR (mmHg)	< 0.001*	< 0.001*	0.003*	0.003*
Diastolic RR (mmHg)	0.007*	< 0.001*	0.003*	< 0.001*
Antihypertensive medication	0.03*	0.001*	0.08*	0.61
Triglyceride levels (mg/dL)	*< 0.001*	*< 0.001*	*0.001*	*< 0.001*
Total cholesterol (mg/L)	0.90	0.003*	0.001*	0.001*
HDL (mg/dL)	*< 0.001*	*< 0.001*	*0.02*	*< 0.001*
LDL (mg/dL)	0.54	*0.001*	*0.001*	*< 0.001*
Lipid lowering medication	*0.05*	*0.02*	0.42	0.46
Smoking status	0.30	0.48	0.98	0.99

Covariates with a p value < 0.10 were included in multivariate analysis (italicized). In case of co-linearity (*), the more common definition of the risk factors was included. Age, sex and BMI were included in the model based on previous literature independent of the p value (PreDef)

**Table 5:** Sensitivity-Analysis of the Multivariate Association Models

	LVM		LVCI		LVEDV		LVSV	
	β (95% CI)	p	β (95% CI)	p	β (95% CI)	p	β (95% CI)	p
Initial analysis (= Table 2): separate, fully^a^ adjusted models as in the main analysis								
VAT	–		0.11 (0.07; 0.15)	< 0.001	− 6.70 (− 8.84; − 4.55)	< 0.001	− 3.91 (− 5.32; − 2.50)	< 0.001
SAT	–		–		–		− 1.75 (− 3.66; 0.16)	0.07
PDFF_hepatic_	–		0.06 (0.02; 0.09)	0.001	− 3.23 (− 5.03; − 1.44)	< 0.001	− 2.20 (− 3.37; − 1.04)	< 0.001
Sensitivity analysis 1: separate, fully^b^ adjusted models with a fixed set of typical cardiovascular risk factors								
VAT	–		0.13 (0.09; 0.16)	< 0.001	− 7.04 (− 9.12; − 4.97)	< 0.001	− 4.38 (− 5.76; − 3.01)	< 0.001
SAT	–		–		–		− 1.66 (− 3.62; 0.29)	0.10
PDFF_hepatic_	–		0.07 (0.03; 0.10)	< 0.001	− 3.54 (− 5.35; − 1.74)	< 0.001	− 2.47 (− 3.66; − 1.29)	< 0.001
Sensitivity analysis 2: separate, fully^c^ adjusted models with a fixed set of typical cardiovascular risk factors and a replacement of the definition of hypertension								
VAT	–		0.12 (0.08; 0.15)	< 0.001	− 6.79 (− 8.88; − 4.7)	< 0.001	− 4.22 (− 5.59; − 2.85)	< 0.001
SAT	–		–		–		− 1.49 (− 3.41; 0.43)	0.13
PDFF_hepatic_	–		0.06 (0.02; 0.09)	0.001	− 3.30 (− 5.13; − 1.48)	< 0.001	− 2.32 (− 3.51; − 1.14)	< 0.001

The main multivariate analysis included potential confounders, selection was based on univariate analysis as detailed in Appendix Table 4 (^a^ the model included age, sex, BMI, hypertension, diabetes, triglycerides, HDL (for all LV parameters), additionally LDL (for LVCI, LVEDV, and LVSV) and lipid lowering medication (for LVM, and LVCI)). In the sensitivity analysis, a model with fixed set of typical cardiovascular risk factors as potential confounders were conducted (^b^ the model included age, sex, BMI, hypertension, diabetes, smoking). Further, the definition of hypertension was replaced by continuous measurements of systolic and diastolic blood pressure and presence if antihypertensive medication (^c^ the fully adjusted model included age, sex, BMI, systolic blood pressure, diastolic blood pressure, antihypertensive medication, diabetes, smoking)

## Abbreviations

BMI: body mass index
BSA: body surface area
CI: confidence intervals
EF: ejection fraction
IFG: impaired fasting glucose
IGT: impaired glucose tolerance
KORA: Cooperative Health Research in the Region of Augsburg
LV: left-ventricular
LVCI: left-ventricular concentricity index
LVEDV: left-ventricular end-diastolic volume
LVEF: left-ventricular ejection fraction
LVESV: left-ventricular end-systolic volume
LVM: left-ventricular myocardial mass
LVSV: left-ventricular stroke volume
NAFLD: nonalcoholic fatty liver disease
OGT: oral glucose tolerance test
PDFF_hepatic_: hepatic proton-density fat fraction
SAT: subcutaneous adipose tissue
WHR: waist-to-hip-ratio

## References

[1] King H, Aubert RE, Herman WH (1998). Global burden of diabetes, 1995–2025: prevalence, numerical estimates, and projections. Diabetes Care.

[2] Nathan DM, Davidson MB, DeFronzo RA, Heine RJ, Henry RR, Pratley R, Zinman B, American Diabetes A (2007). Impaired fasting glucose and impaired glucose tolerance: implications for care. Diabetes Care.

[3] Cowie CC, Rust KF, Byrd-Holt DD, Eberhardt MS, Flegal KM, Engelgau MM, Saydah SH, Williams DE, Geiss LS, Gregg EW (2006). Prevalence of diabetes and impaired fasting glucose in adults in the US population: national health and nutrition examination survey 1999–2002. Diabetes Care.

[4] Danaei G, Lawes CM, Vander Hoorn S, Murray CJ, Ezzati M (2006). Global and regional mortality from ischaemic heart disease and stroke attributable to higher-than-optimum blood glucose concentration: comparative risk assessment. Lancet.

[5] Bamberg F, Hetterich H, Rospleszcz S, Lorbeer R, Auweter SD, Schlett CL, Schafnitzel A, Bayerl C, Schindler A, Saam T (2017). Subclinical disease burden as assessed by whole-body MRI in subjects with prediabetes, subjects with diabetes, and normal control subjects from the general population: the KORA-MRI study. Diabetes.

[6] Malavazos AE, Corsi MM, Ermetici F, Coman C, Sardanelli F, Rossi A, Morricone L, Ambrosi B (2007). Proinflammatory cytokines and cardiac abnormalities in uncomplicated obesity: relationship with abdominal fat deposition. Nutr Metab Cardiovasc Dis.

[7] Pou KM, Massaro JM, Hoffmann U, Vasan RS, Maurovich-Horvat P, Larson MG, Keaney JF, Meigs JB, Lipinska I, Kathiresan S (2007). Visceral and subcutaneous adipose tissue volumes are cross-sectionally related to markers of inflammation and oxidative stress: the Framingham heart study. Circulation.

[8] Schlett CL, Hoffmann U (2011). Identification and quantification of fat compartments with CT and MRI and their importance. Radiologe.

[9] Ammar KA, Redfield MM, Mahoney DW, Johnson M, Jacobsen SJ, Rodeheffer RJ (2008). Central obesity: association with left ventricular dysfunction and mortality in the community. Am Heart J.

[10] Rider OJ, Francis JM, Ali MK, Byrne J, Clarke K, Neubauer S, Petersen SE (2009). Determinants of left ventricular mass in obesity; a cardiovascular magnetic resonance study. J Cardiovasc Magn Reson.

[11] Neeland IJ, Gupta S, Ayers CR, Turer AT, Rame JE, Das SR, Berry JD, Khera A, McGuire DK, Vega GL (2013). Relation of regional fat distribution to left ventricular structure and function. Circ Cardiovasc Imaging.

[12] Capoulade R, Larose E, Mathieu P, Clavel MA, Dahou A, Arsenault M, Bédard E, Larue-Grondin S, Le Ven F, Dumesnil JG (2014). Visceral adiposity and left ventricular mass and function in patients with aortic stenosis: the PROGRESSA study. Can J Cardiol.

[13] Shah RV, Abbasi SA, Heydari B, Rickers C, Jacobs DRJ, Wang L, Kwong RY, Bluemke DA, Lima JA, Jerosch-Herold M (2013). Insulin resistance, subclinical left ventricular remodeling, and the obesity paradox: MESA (multi-ethnic study of atherosclerosis). J Am Coll Cardiol.

[14] Abbasi SA, Hundley WG, Bluemke DA, Jerosch-Herold M, Blankstein R, Petersen SE, Rider OJ, Lima JA, Allison MA, Murthy VL, Shah RV (2015). Visceral adiposity and left ventricular remodeling: the multi-ethnic study of atherosclerosis. Nutr Metab Cardiovasc Dis.

[15] Park J, Kim NH, Kim SH, Kim JS, Kim YH, Lim HE, Kim EJ, Na JO, Cho GY, Baik I (2014). Visceral adiposity and skeletal muscle mass are independently and synergistically associated with left ventricular structure and function: the Korean genome and epidemiology study. Int J Cardiol.

[16] Fox CS, Gona P, Hoffmann U, Porter SA, Salton CJ, Massaro JM, Levy D, Larson MG, D’agostino RB, O’donnell CJ, Manning WJ (2009). Pericardial fat, intrathoracic fat, and measures of left ventricular structure and function: the Framingham heart study. Circulation.

[17] Fontes-Carvalho R, Fontes-Oliveira M, Sampaio F, Mancio J, Bettencourt N, Teixeira M, Rocha Gonçalves F, Gama V, Leite-Moreira A (2014). Influence of epicardial and visceral fat on left ventricular diastolic and systolic functions in patients after myocardial infarction. Am J Cardiol.

[18] Canepa M, Strait JB, Milaneschi Y, AlGhatrif M, Ramachandran R, Makrogiannis S, Moni M, David M, Brunelli C, Lakatta EG, Ferrucci L (2013). The relationship between visceral adiposity and left ventricular diastolic function: results from the Baltimore longitudinal study of aging. Nutr Metab Cardiovasc Dis.

[19] Granér M, Nyman K, Siren R, Pentikäinen MO, Lundbom J, Hakkarainen A, Lauerma K, Lundbom N, Nieminen MS, Taskinen MR (2014). Ectopic fat depots and left ventricular function in nondiabetic men with nonalcoholic fatty liver disease. Circ Cardiovasc Imaging.

[20] Neeland IJ, Turer AT, Ayers CR, Powell-Wiley TM, Vega GL, Farzaneh-Far R, Grundy SM, Khera A, McGuire DK, de Lemos JA (2012). Dysfunctional adiposity and the risk of prediabetes and type 2 diabetes in obese adults. J Am Med Assoc.

[21] Holle R, Happich M, Lowel H, Wichmann HE, Group MKS (2005). KORA—a research platform for population based health research. Gesundheitswesen.

[22] Press W, Organization WH (2006). Definition and diagnosis of diabetes mellitus and intermediate hyperglycemia. Publications of the World Health Organization.

[23] Schwenzer NF, Machann J, Schraml C, Springer F, Ludescher B, Stefan N, Haring H, Fritsche A, Claussen CD, Schick F (2010). Quantitative analysis of adipose tissue in single transverse slices for estimation of volumes of relevant fat tissue compartments: a study in a large cohort of subjects at risk for type 2 diabetes by MRI with comparison to anthropometric data. Invest Radiol.

[24] Wurslin C, Machann J, Rempp H, Claussen C, Yang B, Schick F (2010). Topography mapping of whole body adipose tissue using a fully automated and standardized procedure. J Magn Reson Imaging.

[25] Tang A, Tan J, Sun M, Hamilton G, Bydder M, Wolfson T, Gamst AC, Middleton M, Brunt EM, Loomba R (2013). Nonalcoholic fatty liver disease: MR imaging of liver proton density fat fraction to assess hepatic steatosis. Radiology.

[26] Hetterich H, Bayerl C, Peters A, Heier M, Linkohr B, Meisinger C, Auweter S, Kannengiesser SA, Kramer H, Ertl-Wagner B, Bamberg F (2016). Feasibility of a three-step magnetic resonance imaging approach for the assessment of hepatic steatosis in an asymptomatic study population. Eur Radiol.

[27] Schulz-Menger J, Bluemke DA, Bremerich J, Flamm SD, Fogel MA, Friedrich MG, Kim RJ, von Knobelsdorff-Brenkenhoff F, Kramer CM, Pennell DJ (2013). Standardized image interpretation and post processing in cardiovascular magnetic resonance: society for cardiovascular magnetic resonance (SCMR) board of trustees task force on standardized post processing. J Cardiovasc Magn Reson.

[28] Gaasch WH, Zile MR (2011). Left ventricular structural remodeling in health and disease: with special emphasis on volume, mass, and geometry. J Am Coll Cardiol.

[29] Khouri MG, Peshock RM, Ayers CR, de Lemos JA, Drazner MH (2010). A 4-tiered classification of left ventricular hypertrophy based on left ventricular geometry: the Dallas heart study. Circ Cardiovasc Imaging.

[30] Bang CN, Gerdts E, Aurigemma GP, Boman K, de Simone G, Dahlöf B, Køber L, Wachtell K, Devereux RB (2014). Four-group classification of left ventricular hypertrophy based on ventricular concentricity and dilatation identifies a low-risk subset of eccentric hypertrophy in hypertensive patients. Circ Cardiovasc Imaging.

[31] Patel BN, Kumbla RA, Berland LL, Fineberg NS, Morgan DE (2013). Material density hepatic steatosis quantification on intravenous contrast-enhanced rapid kilovolt (peak)-switching single-source dual-energy computed tomography. J Comput Assist Tomogr.

[32] Cassidy S, Hallsworth K, Thoma C, MacGowan GA, Hollingsworth KG, Day CP, Taylor R, Jakovljevic DG, Trenell MI (2015). Cardiac structure and function are altered in type 2 diabetes and non-alcoholic fatty liver disease and associate with glycemic control. Cardiovasc Diabetol.

[33] Francque SM, van der Graaff D, Kwanten WJ (2016). Non-alcoholic fatty liver disease and cardiovascular risk: pathophysiological mechanisms and implications. J Hepatol.

[34] Adiels M, Taskinen MR, Packard C, Caslake MJ, Soro-Paavonen A, Westerbacka J, Vehkavaara S, Hakkinen A, Olofsson SO, Yki-Jarvinen H, Boren J (2006). Overproduction of large VLDL particles is driven by increased liver fat content in man. Diabetologia.

[35] Pasarin M, La Mura V, Gracia-Sancho J, Garcia-Caldero H, Rodriguez-Vilarrupla A, Garcia-Pagan JC, Bosch J, Abraldes JG (2012). Sinusoidal endothelial dysfunction precedes inflammation and fibrosis in a model of NAFLD. PLoS ONE.

[36] VanWagner LB, Wilcox JE, Colangelo LA, Lloyd-Jones DM, Carr JJ, Lima JA, Lewis CE, Rinella ME, Shah SJ (2015). Association of nonalcoholic fatty liver disease with subclinical myocardial remodeling and dysfunction: a population-based study. Hepatology.

